# Improving Relationships by Elevating Positive Illusion and the Underlying Psychological and Neural Mechanisms

**DOI:** 10.3389/fnhum.2018.00526

**Published:** 2019-01-11

**Authors:** Hongwen Song, Yongjun Zhang, Lin Zuo, Xueli Chen, Gui Cao, Federico d’Oleire Uquillas, Xiaochu Zhang

**Affiliations:** ^1^School of Humanities and Social Science, University of Science and Technology of China, Hefei, China; ^2^School of Foreign Languages, Anhui Jianzhu University, Hefei, China; ^3^Center for Biomedical Engineering, School of Information Science and Technology, University of Science and Technology of China, Hefei, China; ^4^Hefei Medical Research Center on Alcohol Addiction, Anhui Mental Health Center, Hefei, China; ^5^Institute of Health Science Research, School of Sociology and Population Studies, Renmin University of China, Beijing, China; ^6^Department of Neurology, Massachusetts General Hospital, Harvard Medical School, Boston, MA, United States; ^7^Academy of Psychology and Behavior, Tianjin Normal University, Tianjin, China

**Keywords:** positive illusion, romantic love, relationships, commitment, relationship satisfaction

## Abstract

Romantic relationships are difficult to maintain novel and exciting for long periods of time, and individuals in love are known to engage in a variety of efforts to protect and maintain their romantic relationship. How to protect and maintain these relationships more effectively has, however, plagued people, psychologists, and therapists. Intimate partners typically perceive their relationship and their partners in a positive light or bias, a phenomenon called positive illusion. Interestingly, higher levels of positive illusion between partners have been associated with a decreased risk for relationship dissolution, as well as higher satisfaction, and less conflict or doubt in relationships. These findings indicate that elevating positive illusion amongst romantic partners may be of benefit and improve romantic relationships. In the present article, we discuss solving the paradox of positive illusion. As positive illusion may have relationship-enhancing attributes, we discuss the psychological and neural mechanisms that may underlie positive illusion. By elucidating the mechanisms underlying positive illusion, we shine a spotlight on potential future directions for research that aims to improve positive illusion and thus enhance the satisfaction and longevity of romantic relationships.

## Introduction

As one of the most captivating affective states, romantic love has edified some of the loftiest achievements of mankind throughout the ages (Bartels and Zeki, 2000). Early research on love and relationships focused primarily on the description, interpretation, and development of romantic relationships (Le et al., 2010). In the past 20 years, research has gradually shifted towards studying the stability of these romantic relationships.

Will love fade as time goes by? In the initial stage of love, the loving partners indubitably hope to spend the rest of their lives together. However, studies suggest that love may not last forever (Fisher et al., 2016; Zou et al., 2016). For example, scores from lovers on the Passionate Love Scale have been found to decrease over time (Hatfield et al., 2008; McNulty et al., 2017). While many experience gradual decreases in their perception of romantic love towards their partners, it may not all be doom and gloom for everybody. For example, a study conducted by Acevedo et al. (2012) revealed that individuals in long-term relationships (marriages lasting an average of 21.4 years) showed similar brain activation patterns while viewing facial images of their partners as individuals reporting to have recently fallen in love (length of time in love about 3–18 months). This begs the question of what factors influence the maintenance of romantic relationships. Le et al. (2010) believe that a construct called “positive illusion” is the best predictor for the maintenance of subjective feelings of romantic love (Cohen’s *d*: −0.991), in comparison to other relationship-related variables such as commitment, love, and satisfaction. This finding highlights that cognitive processing and biases play a significant role in stay-leave decisions regarding romantic relationships (Le et al., 2010), and offer a potentially promising avenue for the study and intervention of romantic relationship maintenance.

In recent years, researchers have posited that a positive illusion about their partner may be closely related to relationship satisfaction and relationship persistence (Miller et al., 2006; Barelds and Dijkstra, 2011; Abbasi, 2017; Ogolsky et al., 2017). Positive illusion is usually defined as a tendency to perceive one’s own relationship as having higher positive characteristics and lesser negative characteristics than other people’s relationships (Murray et al., 1996a), or a tendency to view one’s own romantic partner more favorably than their partner views themself (Neff and Karney, 2002). Additionally, a higher positive illusion level during the initial stages of a relationship may predict a subsequent slower decline in relationship satisfaction over time, as well as a greater likelihood of relationship persistence (Murray and Holmes, 1997; Murray et al., 2011; Finkel et al., 2013; Dijkstra et al., 2014). While previous studies suggest that an elevated positive illusion regarding their partner might benefit and improve the length of romantic relationships, researchers have yet to discover ways of enhancing this predictive marker of romantic love. In the present article, we discuss solving the paradox of positive illusion that may have a relationship-enhancing function. Furthermore, we discuss the potential psychological and neural mechanisms behind positive illusion. Based on the discussion regarding the potential mechanisms that may underlie positive illusion, we assert several potential directions for future research on this topic, specifically, how to improve positive illusion in romantic relationships.

## Solving the Paradox of Positive Illusion

Social psychology researchers have studied positive illusion in relationships for decades now, and have debated whether positive illusion is a key feature that defines the well-being of an enduring relationship, as well as whether a lack thereof can leave people vulnerable toward disillusionment and regretful decision-making (Fletcher, 2015; Abbasi, 2017). While some argue that positive illusion is crucial for dating and marital relationship satisfaction (Martz et al., 1998), others view positive illusion as an unpropitious mental disorder akin to addiction (Brickman et al., 1987). However, there is significant evidence supporting the former view. For example, individuals with higher positive illusions of their relationship enjoy higher relationship satisfaction, less conflicts and doubts, and decreased risk for relationship discontinuation (Barelds and Dijkstra, 2009, 2011; Le et al., 2010; Abbasi, 2017). From an evolutionary perspective, romantic love is often seen as a commitment mechanism that promotes the nurture of offspring by encouraging couples to engage in a substantial investment (Fletcher et al., 2015). The ultimate realization of long-term love commitments requires a leap of faith that manifests as positive illusion to quell doubt and produce a sense of security (Murray and Holmes, 1997). However, the Darwinian perspective suggests that mate-selection criteria for any species has evolved based on natural and sexual selection (Darwin, 1888). According to this view, human mate-selection must rest on reasonably accurate judgments about physical attractiveness, and the status of potential partners (Fletcher, 2015). Thus, these differing arguments produce a paradox in that individuals commit to long-term relationships based on both objective and subjective judgments.

To attempt to reconcile this paradox, researchers have conducted a series of experiments. Rusbult et al. (2000) for example, decreased, to some extent, individuals’ positive illusion about their partner by manipulating the experimental instructions by which participants were required to describe their own relationship as honestly and accurately as possible. Although in the accurate instructions condition, positive illusion was slightly reduced rather than completely eliminated, this was the first attempt to separate illusion from reality, and the first tentative solution to the evolutionary paradox. Positive illusion, thus, does not seem to be more blind than prescient (Murray et al., 1996b), and Gagné and Lydon (2004) believe that one can be both biased (holding positive illusions) and accurate at the same time, as accuracy and positive illusion may coexist in people’s evaluations of their relationships. Positive illusion can provide a constant sense of security, regulate feelings regarding a relationship, and help maintain faith that a relationship is worth pursuing (Murray and Holmes, 1999). On the other hand, accuracy helps avoid future disillusionment and regretful decision making (Gagné and Lydon, 2004; Fletcher, 2015).

Overall, positive illusion motivates individuals to perceive their partners or relationships in a realistic positive light. It influences individuals to interpret the shortcomings of their partner in a kind and generous manner rather than to directly ignore those shortcomings. Importantly, people with positive illusion do not tend to attribute false desirable characteristics to their partners (Luo et al., 2010). In summary, as time goes by, positive illusion is associated with greater relationship satisfaction, care, trust, and lasting intimacy—hallmarks of healthy relationships.

## Relationship-Enhancing Function of Positive Illusion

Relationships are not all smooth-sailing, and we are often confronted by a variety of unavoidable issues that constantly challenge the stability of our relationships. Sometimes we overcome these issues with an optimistic mentality; while at other times we confront such challenges in a negative manner. Compared with a pessimistic mentality, optimism is more likely to yield beneficial results (Miller and Turnbull, 1986). As a defined pattern of optimistic belief, how does positive illusion help confront threats to relationships and address challenges?

Positive illusion can help with facing inevitable threats to relationships. Most relationships are inevitably threatened by conflicts of interest or seductive alternatives, and solving such problems often requires a departure from one’s own direct interests. For example, when a partner behaves badly, accommodation rather than revenge are more conducive to the stability of the relationship (Rusbult et al., 1991; Van Lange et al., 1997). Further, when partners’ preferences are inconsistent, it is beneficial to sacrifice one’s own interests for the partner’s interests. Overall, positive belief systems motivate us to find available solutions to dilemmas found across relationships (Murray et al., 1996a). Such systems promote persistence, by increasing pro-social motivation, and facilitating a willingness to investment oneself in a relationship (Miller et al., 2006; Le et al., 2010). Thus, it is plausible that positive illusion may serve to enhance the health of relationships.

Positive illusion may also help sustain faith in relationships when there is uncertainty or doubt. Even the most idyllic relationships suffer from difficult periods that evoke feelings of discontent or suspicion. It is believed, however, that positive illusion may reduce suspicion or uncertainty from potentially confounding information (Niehuis et al., 2011).

Positive illusion may also maintain a relationship by improving self-esteem, and has been shown to be associated with self-fulfillment. For example, the idealization between partners can promote self-fulfillment that immunizes intimates against the detrimental effects of early suspicion and conflict, thereby enhancing later satisfaction (Murray et al., 1996b; Fletcher, 2015; Erol and Orth, 2016). From this perspective, individuals that hold positive beliefs about their relationship will often feel greater satisfaction about themselves, and this has been shown to make it more likely for their relationship to persist (Boyes and Fletcher, 2007; Barelds and Dijkstra, 2009; Erol and Orth, 2014).

In summary, positive illusion has a relationship-enhancing function that can buffer conflicts and doubts, enhance the maintenance of relationships, increase the level of relationship satisfaction through the application of coping mechanisms to inevitable challenges, and foster an improvement in a partner’s self-esteem.

## Psychological Mechanisms of Positive Illusion

In previous sections, we discussed the relationship-enhancing function of positive illusion. For such an important phenomenon, we will next discuss how it may be generated and maintained from the perspective of psychological mechanisms.

As to the generation of positive illusion, previous studies have raised two points of view: (1) Murray et al. (1996a) suggested that in close relationships, people may project their own virtues and their ideal partners’ virtues onto their current partners; and (2) Commitment is a motivator of positive illusion. When individuals invest a higher level of commitment in their relationship, they also take a more favorable view towards their relationship (Gagné and Lydon, 2004). Although some empirical studies partially support these views, many questions remain unclear. With regard to the first view, researchers have used cross-sectional data to study the causal link among individuals’ self-images, ideals, and the impressions of their partners (Murray et al., 1996a), and their results suggested that the impressions of their partners were a mirror of individuals’ self-images and ideals. However, the characteristics of a current partner may confound that individual’s criteria of their ideal partner, and thus, measuring the development of positive illusion via cross-sectional data is quite limited and requires further longitudinal research. With regard to the second view, Rusbult et al. (2000) used threatening instructions to manipulate the level of commitment, in an attempt to study the motivator of positive illusion. Their results suggested that manipulating commitment leads to only a partial change rather than a complete elimination of positive illusion. This result, thus, did not provide direct evidence in support of a causal link between commitment and positive illusion. An association between commitment and positive illusion might fit into a model of cyclical growth in which variables represented as “later effects” feed back into and influence “earlier causes.” Therefore, the researchers ultimately did not seem to agree on how positive illusion arises, and this requires additional studies to further clarify their results.

Why does positive illusion persist, even in the face of conflicting information? Compared to the development of positive illusion, the maintenance of positive illusion is even more essential. Indeed, some individuals’ positive perceptions about their partners do not disappear, but instead, become more prominent as time goes by Miller et al. (2006). Researchers have suggested that social comparison includes dimensional comparison (selectively focusing on advantaged dimensions of one’s own relationship), downward comparison (comparing others’ relationships that are worse off), avoidance of comparison (ignoring information that is detrimental to one’s own relationship), and the manipulation of surrounding dimensions (selectively focusing on information that derogate others’ relationship), all crucial for developing and maintaining positive illusion (Wood and Taylor, 1991). Furthermore, Murray et al. (1996a) believed that self-images play an important role in structuring the images of others, and found that more positive self-images contribute to the maintenance of positive perceptions of partners. Although the above-mentioned studies have tried to assess how positive illusion is maintained, thus far, there is still no empirical research that has been able to study the mechanisms that underlie the maintenance of positive illusion.

## Neural Mechanism of Positive Illusion

**Figure 1:**
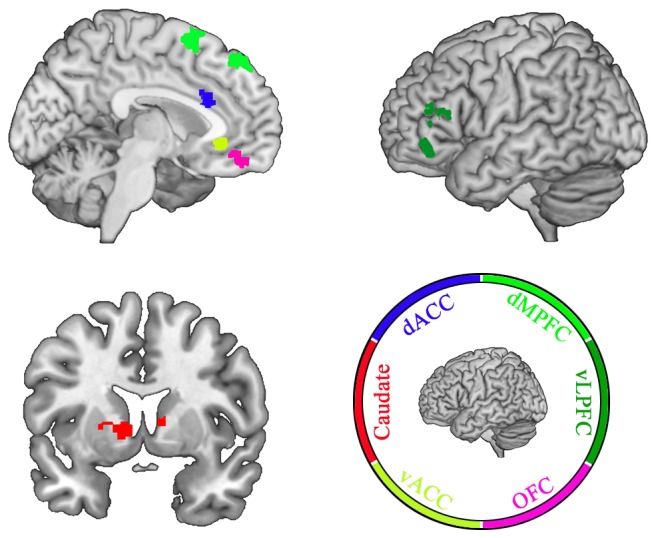
Brain areas related to positive illusion. This image is an original schematic diagram, and the brain areas referred to previous studies (Meyer et al., 2011; Hughes and Beer, 2012). Definitions: dACC, dorsal anterior cingulate cortex; dMPFC, dorsal medial prefrontal cortex; vLPFC, ventrolateral prefrontal cortical regions; OFC, the orbitofrontal cortex; vACC, ventral anterior cingulate cortex.

With the advent of magnetic resonance imaging (MRI) technology, researchers have become increasingly interested in investigating the underlying neural mechanisms of positive illusion. Recent functional MRI (fMRI) research has revealed the following brain regions as being associated with positive illusion (see Figure 1): (a) the caudate nucleus; (b) the dorsal anterior cingulate cortex (dACC); (c) the ventral anterior cingulate cortex (vACC); (d) the orbitofrontal cortex (OFC); (e) the ventrolateral prefrontal cortical regions (vLPFC); and (f) the dorsal medial prefrontal cortex (dMPFC; Meyer et al., 2011; Hughes and Beer, 2012). Interestingly, these areas play important roles in the processing of reward (the caudate nucleus; Aron et al., 2005), subjective valuation (OFC; De Martino et al., 2006; Fellows, 2007), conflict detection (dACC; Botvinick et al., 2004; Kawamoto et al., 2012), and emotional control (vACC, vLPFC, and dMPFC; Meyer et al., 2011).

The caudate nucleus is a dopaminergic brain area associated with goal-directed motivation and reward, and is usually considered as one of the neurobiological correlates of romantic love (Aron et al., 2005). Activity found here has been associated with the perception of a romantic partner (i.e., this region activates upon looking at a photograph of a romantic partner) as compared with familiar friends (Bartels and Zeki, 2000; Aron et al., 2005; Xu et al., 2011). Importantly, the caudate nucleus is connected to the ventral tegmental area (VTA) and nucleus accumbens (NAC) by dopaminergic neurons, and is associated with the processing of love-related reward signals that drive individuals to approach a romantic love target (Lauwereyns, 2006). It is believed that the activation of the caudate nucleus lends salience to the positive characteristics of a romantic partner over negative characteristics or other social comparisons (Hughes and Beer, 2012).

The dACC has been associated with error detection, monitoring of conflict, and social exclusion (Botvinick et al., 2004; Kawamoto et al., 2012). Participants in experiments display significantly elevated activation of the dACC when experiencing negative social evaluation and social exclusion (Eisenberger et al., 2011; Kross et al., 2011). Therefore, a reduction of dACC activation in response to partner-related negative information may represent an adaptive response to a partner’s imperfections.

The vACC has been shown to play a key role in emotion conflict regulation and emotion control (Etkin et al., 2011). Increased activation of the vACC could enhance the differentiation of desirable social characteristics between intimate and non-intimate individuals (Hughes and Beer, 2012).

The OFC has been associated with the encoding of subjective value and the weighing of information (positive or negative) in decision-making (De Martino et al., 2006; Fellows, 2007). Social perception of ordinary individuals (i.e., the average peer), represents the integration of both desirable and undesirable information about an evaluation target. In social comparisons, the positive information about a potential partner becomes available and is integrated into the target personality characteristics of a partner (Kunda, 1990; Rusbult et al., 2000). In theory, the OFC may modulate priority integration of positive information (Hughes and Beer, 2012). The vLPFC and dMPFC are part of a brain network responsible for deliberate emotion regulation (i.e., top-down control of emotional responses; Ochsner et al., 2009). The activation of the vLPFC and dMPFC may benefit the suppression of affective responses that help attract or derogate alternatives (Meyer et al., 2011).

**Figure 2:**
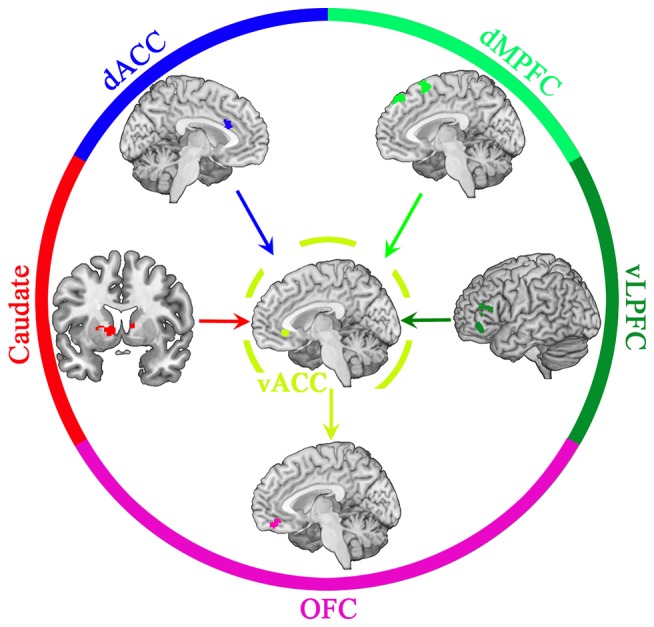
Theoretical schematic diagram of signal processing during generation of positive illusions.

Importantly, these brain areas do not function independently, but rather interact with one other, a mechanism suggested by functional connectivity among these areas (i.e., synchronous activation between these distal brain regions; Cohen et al., 2005; Turner et al., 2006; Greicius et al., 2007; Zald et al., 2012; Song et al., 2015). Taken into context, the comparison between partners and non-close others may be facilitated and made more prominent by the processing of a partner’s positive characteristics by the caudate nucleus, while the dACC suppresses the perception of a partner’s negative features. At the same time, the vLPFC and dMPFC could be reducing the salience of attractive alternatives. Subsequently, these brain areas transmit signals to the vACC that may help differentiate information from potential partners over non-close others. Finally, as information is passed onto the OFC, the weighing of positive and negative information of a partner is redistributed, cementing biased subjective values (e.g., positive illusion) about the partner (see Figure 2).

## Summary and Future Directions

In this article, we discussed solving the paradox of positive illusion. We then reviewed how positive illusion can enhance relationship satisfaction, and longevity. Lastly, we discussed the psychological and neural mechanisms that may underlie positive illusion. While these discussions have deepened our understanding of positive illusion, some questions still remain unresolved.

First, although previous studies have discussed the development of positive illusion, researchers have yet not reached a consensus on this process. As previous studies were unable to provide direct causal evidence, future studies using longitudinal data and strict laboratory experimental manipulations are in need to help elucidate this process.

Second, a better understanding of how individuals maintain positive illusions about their partners when confronted with information inconsistent with previous impressions is essential, as it may help explain the apparent contradiction between reinforcement and environmental fitness (e.g., the evolutionary paradox). Furthermore, this avenue of research may help design interventions that improve positive illusion, as well as help inform future experiment designs that simulate the maintenance of positive illusion and thus reveal potential mechanisms underlying positive illusion maintenance.

Third, while preliminary studies on the neural mechanisms of positive illusion indicate brain activation patterns associated with positive illusion toward romantic partners, studies have yet to explore the neural mechanisms associated with the generation and maintenance of positive illusion. In particular, fMRI experiments investigating how individuals maintain positive illusions of their partner when confronted with information that is inconsistent with previous impressions, remain to be performed. Even further, based on previous knowledge on associated brain areas and networks involved in positive illusion, future research may wish to use non-invasive neural intervention techniques, such as transcranial direct current stimulation (tDCS) or transcranial magnetic stimulation (TMS), to intervene in the generation and maintenance of positive perceptions amongst romantic partners. These techniques manipulate signal processing and brain mechanisms, and are thus elegant ways of investigating causal associations regarding positive illusion and other factors of relationships such as relationship satisfaction, commitment, and conflict resolution.

## Author Contributions

HS and XZ conceived and wrote the frame design. HS wrote the manuscript. HS, XZ, YZ, LZ, FU, XC and GC revised the manuscript, and all authors contributed to the final version.

## Conflict of Interest Statement

The authors declare that the research was conducted in the absence of any commercial or financial relationships that could be construed as a potential conflict of interest.
